# EVACUATING OR SHELTERING IN PLACE DURING A DISASTER: HOW ASSISTED LIVING ADMINISTRATORS MAKE THE DECISION

**DOI:** 10.1093/geroni/igz038.2572

**Published:** 2019-11-08

**Authors:** Lindsay J Peterson, Kathryn Hyer, David Dosa, Joseph June, Debra J Dobbs, Kali S Thomas

**Affiliations:** 1 University of South Florida School of Aging Studies, Tampa, Florida, United States; 2 University of South Florida, Tampa, Florida, United States; 3 Brown University School of Public Health, Providence, Rhode Island, United States; 4 School of Aging Studies, University of South Florida, Tampa, Florida, United States; 5 Providence VA Medical Center, Providence, Rhode Island, United States

## Abstract

The decision to evacuate or shelter in place during a natural disaster such as a hurricane is complicated and poses risks to long-term care residents. While research has documented the difficulty of the evacuation decision for nursing home administrators, little is known about how assisted living residence (ALR) administrators make this decision. This is a concern given the physical and cognitive impairment level of many ALR residents, the increasing number of ALRs in the U.S., and the frequency of natural disasters. The purpose of this paper was to explore the factors that influenced whether assisted living administrators evacuated their ALRs for Hurricane Irma, a large hurricane that made landfall on Florida’s Southwest coast in September, 2017. This qualitative study used semi-structured interviews and focus groups with ALR owners or administrative staff (N=60) with questions including how they prepared for Hurricane Irma, their experiences during the hurricane, including whether they evacuated or sheltered in place, and lessons learned. The sample includes small (< 25 beds) and large ALRs in the multiple Florida counties affected by the hurricane. A content analysis approach was used. Atlas.ti version 7 was used for initial and axial coding. Prevalent themes included “emergency management planning”, “logistics”, “pressure”, “storm characteristics,” and “staffing”. The results of this study have implications for long-term care policy and training, potentially leading to changes in how ALR leaders prepare for and respond to disasters to improve the safety of residents.

